# Combined Correction of Coronal and Rotational Deformities of the Femur With Distal Femoral Osteotomy Using Patient-Specific Instrumentation

**DOI:** 10.1177/03635465251314868

**Published:** 2025-02-05

**Authors:** Georgios Neopoulos, Lukas Jud, Lazaros Vlachopoulos, Sandro F. Fucentese

**Affiliations:** † Balgrist University Hospital, Department of Orthopedics, University of Zurich, Zurich, Switzerland; Investigation performed at Balgrist University Hospital, University of Zurich, Zurich, Switzerland

**Keywords:** distal femoral osteotomy, patellofemoral instability, torsional deformity, coronal deformity, patient-specific instrumentation

## Abstract

**Background:**

Distal femoral osteotomy (DFO) can be used to simultaneously correct coronal and rotational deformities. Patient-specific instruments (PSIs) are known to be helpful in such complex osteotomies, but data on surgical accuracy for the combined correction of coronal and rotational deformities of the femur are missing.

**Purpose:**

To investigate the radiological results of DFO for simultaneous correction of coronal and rotational deformities using PSIs.

**Study Design:**

Case series; Level of evidence, 3.

**Methods:**

All included patients underwent DFO (34 patients, 36 knees) using PSIs for combined correction of coronal and rotational deformities. The hip-knee-ankle angle (HKA) was measured in weightbearing long-leg radiographs, and the femoral torsion was assessed using computed tomography scans, both pre- and postoperatively. The achieved corrections of HKA and femoral torsion were determined for each knee, and surgical accuracy was calculated.

**Results:**

HKA and femoral torsion changed significantly from preoperatively to postoperatively (from 2.4° ± 3.6° vs 0.1° ± 1.8° [*P* < .001] and 31.2° ± 17.2° vs 18.7° ± 7.4° [*P* < .001]). The difference from planned to achieved correction was statistically greater for HKA (–2.9° ± 3.8° vs –2.3° ± 3.5°; *P* = .018) than for femoral torsion (–12.4° ± 11.8° vs –12.3° ± 12.2°; *P* = .771), which did not reach significance. The accuracies of HKA and femoral torsion correction were 1.1° ± 1° and 2.4° ± 1.9°, respectively.

**Conclusion:**

Coronal and rotational deformities of the femur can accurately be corrected simultaneously by a DFO, utilizing PSIs. High accuracy was achieved for the correction of both coronal and rotational deformities, with absolute mean differences from planned to achieved correction of 1.1° and 2.4°, respectively.

Varus or valgus distal femoral osteotomy (DFO) is a reliable treatment option for patients with a coronal deformity in the femur and concomitant unicompartmental knee osteoarthritis or overload.^6,15,31^ Besides correction of varus or valgus deformity, DFO can be used to address femoral rotational deformity, which is frequently seen in patients evaluated with patellofemoral instability.^4,19,26,35^

However, some patients experience both coronal and rotational deformities of the femur. In such cases, instead of 2 separate osteotomies, an appropriate DFO can be performed to correct both deformities simultaneously. The feasibility of coronal and rotational correction by a DFO was demonstrated in 2018 in an experimental cadaveric study.^14^ A recent clinical study showed improved patient-reported outcomes and adequate correction of coronal and rotational deformities by conventionally performed DFO in 14 knees with patellofemoral instability.^11^ However, detailed data on the accuracy of the planned and achieved corrections have not been reported. Notably, there are limited studies available on this topic, most likely because of the complexity of such osteotomies, which require appropriate preoperative planning and precise surgical execution. One technique that has been shown to enhance surgical planning and execution in complex osteotomies is the use of preoperative 3-dimensional (3D) planning, combined with the intraoperative use of patient-specific instruments (PSIs).^1,8,22,23^ PSIs have been proven to be safe surgical tools offering the advantages of improved surgical accuracy, shorter operating times, shorter learning curves, and decreased fluoroscopy time compared with the conventional technique. However, these benefits have so far only been demonstrated for isolated coronal realignment or isolated rotational DFO, as well as correction osteotomies around the pelvis.^2,3,8,17,23,28^

The aim of the present study was to investigate the radiological results of DFO for combined correction of both coronal and rotational deformities using PSIs. Planned and achieved coronal and rotational corrections were compared. We hypothesized that a high accuracy could be achieved in DFO, using PSIs, for the combined correction of coronal and rotational deformities.

## Methods

### Study Design and Patient Selection

The local ethics committee approved this retrospective case study (Zurich Cantonal Ethics Commission, BASEC-Nr. 2023-00389), and informed consent was obtained from all patients. The research was carried out entirely at the authors’ institution, with data extracted from patient records postoperatively. The records of all patients who underwent a DFO, using PSIs, for the combined correction of coronal (either varus or valgus) and rotational (either antetorsion or retrotorsion) deformities from September 2017 until October 2023 at our institution were retrospectively reviewed. Inclusion criteria were patients with (1) available weightbearing long-leg radiographs (LLRs) preoperatively and at 4.5 months postoperatively and (2) available pre- and postoperative computed tomography (CT) data. Exclusion criteria were patients with (1) proximal femoral derotational osteotomies or concomitant tibial osteotomy and (2) previous bony procedures on the affected femur.

### Surgical Planning and Technique

**Figure 1.**
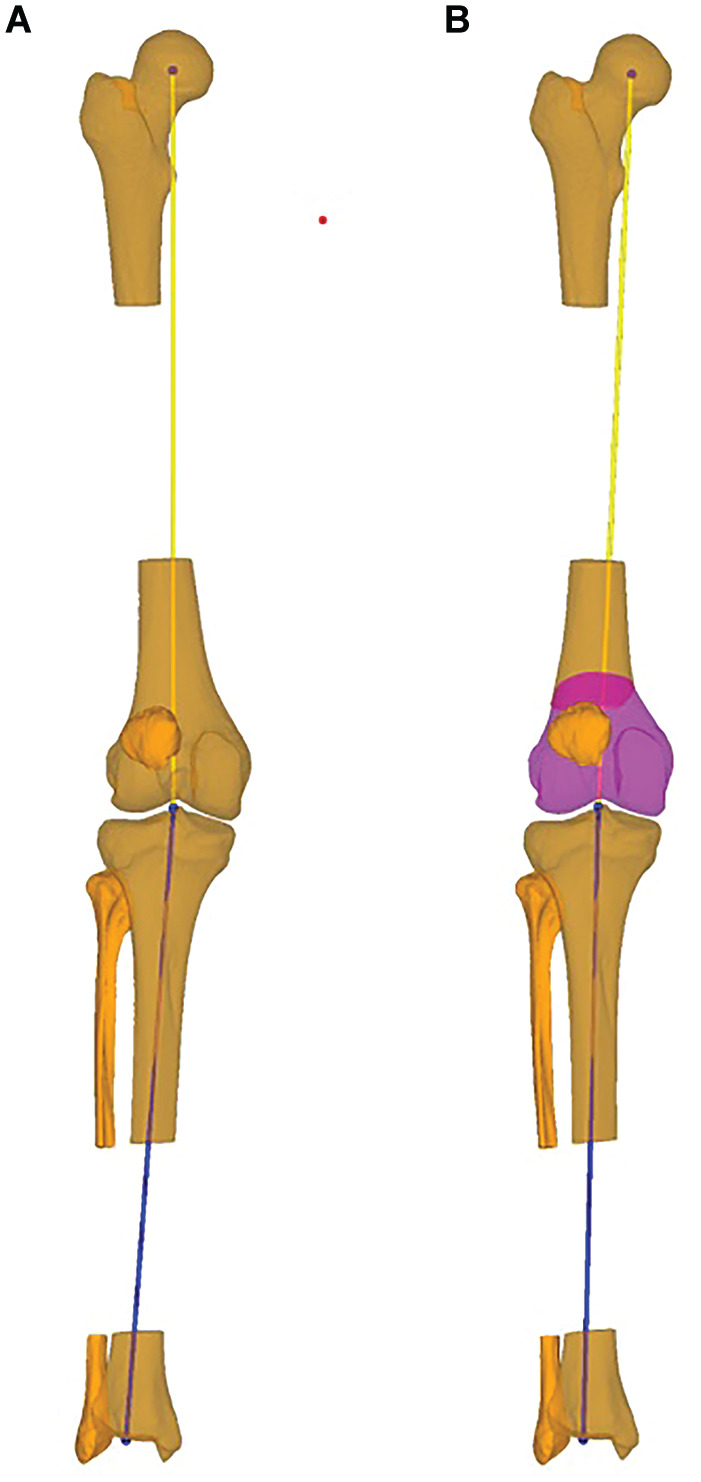
Planned correction of the coronal alignment in 3-dimensional (3D) bone models. Coronal plane of the 3D bone models showing the (A) preoperative and (B) planned coronal alignment of the right leg. The yellow line determines the mechanical axis of the femur, and the blue line the mechanical axis of the tibia. In panel B, the magenta shading represents the distal femur, distal to the osteotomy for better visualization of the osteotomy.

**Figure 2.**
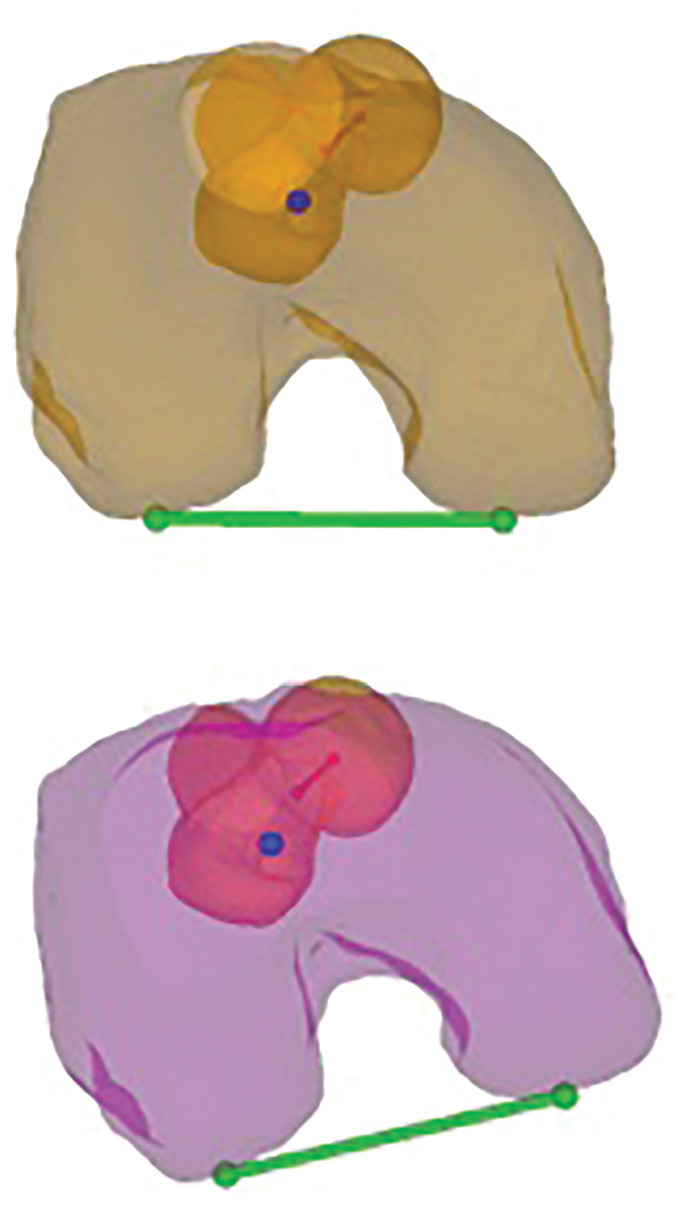
Planned correction of the rotational deformity in 3-dimensional (3D) bone models. Axial plane of the 3D bone models showing the preoperative (orange) and planned (magenta) torsion of the right femur. The bright orange and the bright magenta shading indicate the proximal femur, while the faded orange and faded magenta shading show the distal femur, distal to the osteotomy. The green line determines the posterior condyle axis of the distal femur, and the red line the neck axis of the proximal femur.

**Figure 3.**
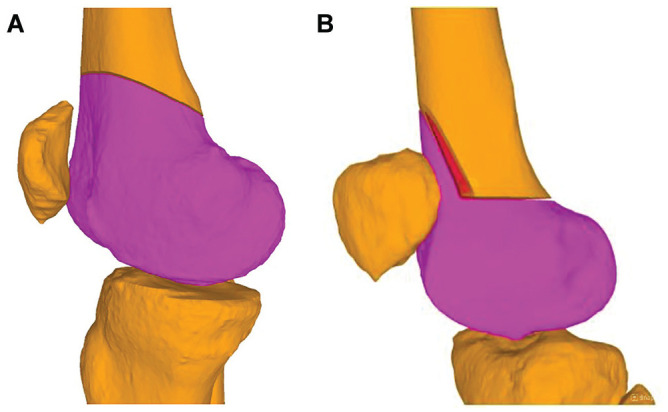
Three-dimensional bone models showing the preoperative planning of the (A) single-cut and (B) biplanar techniques for correction of both rotational and coronal deformities. Shown in red is the wedge that has been removed for closing the biplanar osteotomy.

**Figure 4.**
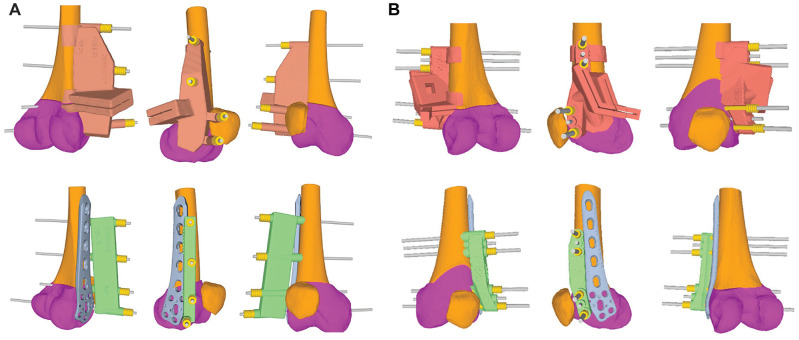
Patient-specific instruments (PSIs) for the distal femoral osteotomy. In the upper row, the prereduction PSIs are shown in beige for (A) single-cut and (B) biplanar distal femoral osteotomies, including the oblique cutting slit alongside the reference pins (gray). In the lower row, the postreduction PSIs are shown in green, including the same reference pins to verify accurate reduction and reposition. The gray TomoFix lateral distal femur plate is positioned next to the postreduction PSI. The magenta shading represents the distal femur, which was repositioned after the osteotomy.

For preoperative planning, triangular surface models of the lower extremities were generated, using CT data. The bone models were subsequently imported into the computer-aided design surgical planning software CASPA (Balgrist CARD AG). Correction of the coronal and rotational deformities was mostly planned to normal values (ie, femoral anteversion of 12° ± 10°, hip-knee-ankle angle [HKA] between 3° of varus and 1° of valgus).^27,29,30^ However, in some cases, the treating surgeon adapted the planning based on individual patient factors (Figures 1 and 2). All surgeries were performed using PSIs. A lateral subvastus approach was used to expose the distal femur. After exposure, if feasible, a biplanar (step-cut) DFO was chosen to correct both coronal and rotational deformities. To achieve the corresponding correction, a wedge is removed in these cases, as shown in Figure 3. However, in cases in which the osteotomy plane for the simultaneous correction of the coronal and rotational deformities had to be planed very steep to achieve the desired correction, a single-cut DFO was chosen to avoid a very proximal directed osteotomy because of the anterior L-shaped osteotomy, as previously described^14^ (Figure 3). Fixation was achieved using mostly a TomoFix lateral distal femur plate (DePuy Synthes). Based on the preoperative 3D planning, if the TomoFix plate did not fit or the bone was too small, an alternative plate was used. The detailed preoperative osteotomy planning, PSI generation (Figure 4), and surgical technique are described in a previous publication.^23^

### Radiological Assessment

**Figure 5.**
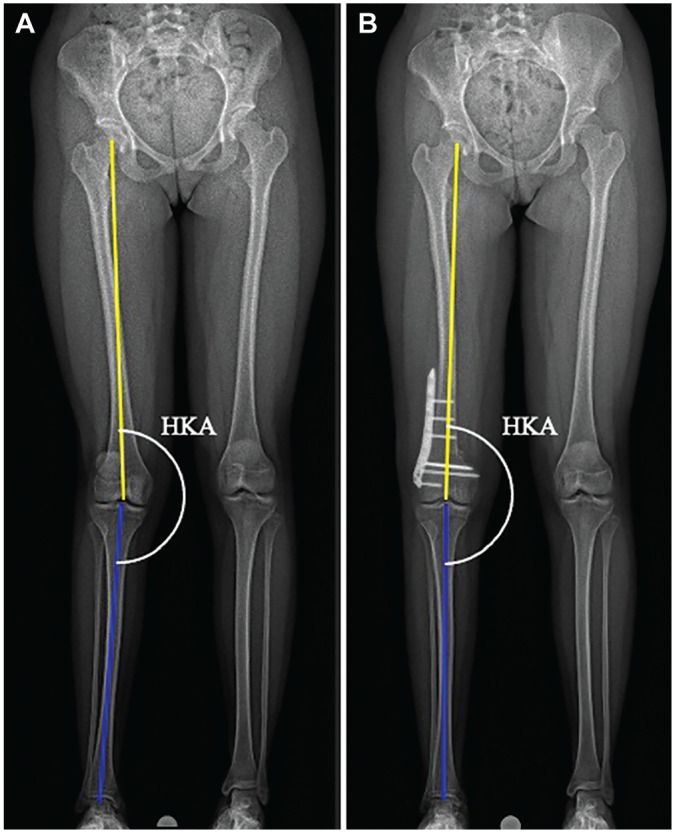
Weightbearing (A) preoperative and (B) postoperative long-leg radiographs showing measurement of the hip-knee-ankle angle (HKA) of the right leg. The top line determines the mechanical axis of the femur, and the bottom line the mechanical axis of the tibia.

**Figure 6.**
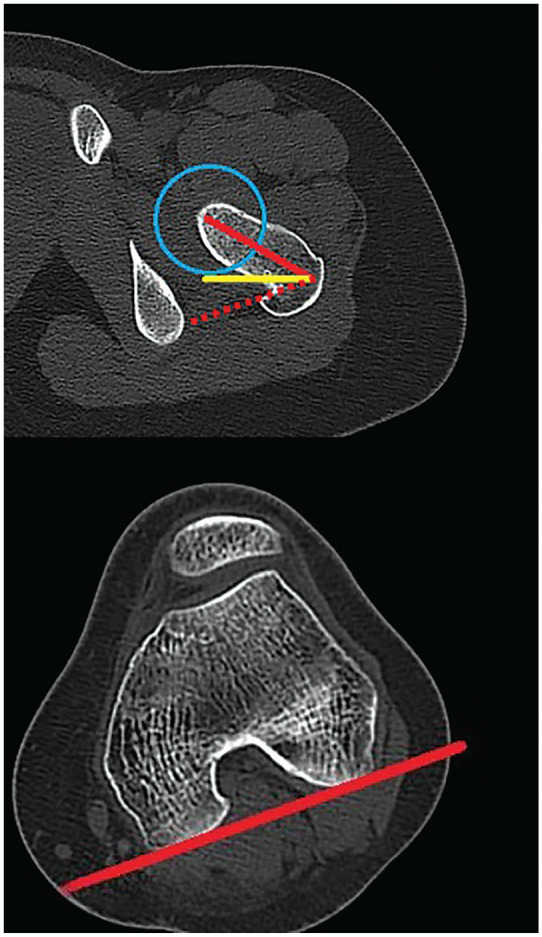
Measurement of femoral torsion in preoperative computed tomography scans of a left (upper) proximal femur and (lower) distal femur in the axial plane. The center of the femoral head was defined. The red line in the upper scan shows the femoral neck axis. The yellow line in the upper scan determined the horizontal plane. The red line in the lower scan and the dotted line in the upper scan represent the parallel line to the posterior femoral condyles. The angle between the dotted line and the red line in the upper scan represents the femoral torsion.

For the coronal alignment, the HKA was measured in the preoperative LLR and at 4.5 months postoperatively (Figure 5).^29,33^ Positive values of the HKA demonstrated a valgus deformity, while negative values indicated a varus deformity. The femoral torsion was measured using the method described by Waidelich et al^34^ in preoperative and 4.5 months postoperative CT data (Figure 6). Negative values indicate femoral retrotorsion, whereas positive values indicate femoral antetorsion.

### Statistical Analysis

Descriptive statistics were utilized to summarize the demographic data of the patients. Continuous variables are reported as mean and standard deviation. The normality of the data distribution was assessed using the Shapiro-Wilk test. Depending on the normality of the data distribution, either the paired *t* test or the Wilcoxon signed-rank test was applied to evaluate differences between pre- and postoperative measurements and between planned and achieved corrections. Achieved corrections of HKA and femoral torsion were calculated for each knee as postoperative HKA/femoral torsion – preoperative HKA/femoral torsion. For the calculation of surgical accuracy, the absolute values of the difference of planned (values from 3D planning) and achieved correction were used. All statistical analyses were performed in SPSS for Windows (Version 29.0; IBM Corp).

## Results

In total, 36 knees (20 left knees, 16 right knees) from 34 patients (27 female, 7 male) were included in this study. The mean age at the time of surgery was 26.1 ± 11.6 years (range, 14-67 years), and the mean body mass index (BMI) was 24.6 ± 5.2 (range, 17.4-38). Detailed patient characteristics are presented in Table 1, and the indications and concomitant procedures performed are shown in Table 2.

**Table 1:** Patient Characteristics*^a^*

Characteristic	Value
No. of knees/patients	36/34
Age, y	26.1 ± 11.6
Female sex	28 (78)
Left side	20 (56)
BMI	24.6 ± 5.2

a Values are presented as No. of knees (%) or mean ± SD. BMI, body mass index.

**Table 2:** Indications and Concomitant Procedures Performed*^a^*

	No. of Knees
Indications	
Patellofemoral instability	28
Patellofemoral maltracking	3
Degenerative	5
Concomitant procedures	
MPFL reconstruction	23
Trochleoplasty	17
Lateral lengthening	20
Tibial tubercle osteotomy	7

a MPFL, medial patellofemoral ligament.

In terms of the osteotomy technique, 20 were performed using a single-cut and 16 a biplanar technique. Patients were restricted to 15 kg of partial weightbearing for 6 weeks; after that, increasing weightbearing was allowed to achieve full weightbearing by 10 to 12 weeks. Radiographs and CT scans obtained at the 4.5-month follow-up confirmed bone union in all 36 knees.

Regarding the coronal deformity, 8 knees required a valgus-producing osteotomy, and 28 knees a varus-producing osteotomy. Pre- and postoperative HKA measurements are summarized in Table 3. Regarding the rotational deformity, 5 knees required an increase, and 31 knees required a decrease in femoral anteversion. Pre- and postoperative measurements of femoral torsion are also summarized in Table 3.

**Table 3:** Pre- and Postoperative Femoral Torsion and HKA*^a^*

	Preoperative	Postoperative	*P* Value
Femoral torsion, deg*^b^*	31.2 ± 17.2 (–20.7 to 52)	18.7 ± 7.4 (4 to 32.5)	<.001
HKA, deg*^c^*	2.4 ± 3.6 (–5.6 to 9)	0.1 ± 1.8 (–3.9 to 4.3)	<.001

a Values are presented as mean ± SD (range). HKA, hip-knee-ankle angle.

b Positive values represent anteversion.

c Positive values represent valgus.

A statistically significant difference was observed between the planned and achieved corrections of the HKA (*P* = .018), whereas there was no statistically significant difference between the planned and achieved corrections of the femoral torsion (*P* = .771). In terms of coronal correction, 20 cases (56%) had <1° deviation between planned and achieved corrections, with 9 cases (25%) hitting the target exactly. In 7 cases (19%), the deviation exceeded 2° but remained below 4°, whereas in 9 cases (25%) the deviation was between 1° and 2°. For rotational correction, 6 cases (17%) had a deviation ≥5° from the planned correction, while the remaining 30 cases (83%) demonstrated a deviation <5°, and 20 cases (56%) had a deviation ≤2°. An overview of planned and achieved corrections is presented in Table 4.

**Table 4:** Planned and Achieved Femoral Derotation as Well as Coronal Correction*^a^*

	Planned	Achieved	*P* Value
Femoral derotation, deg	–12.4 ± 11.8 (–27 to 26.1)	–12.3 ± 12.2 (–26.4 to 30.9)	.771
Coronal correction, deg	–2.9 ± 3.8 (–11 to 4.0)	–2.3 ± 3.5 (–11.5 to 5)	.018

a Values are presented as mean ± SD (range).

The accuracy of HKA correction, in terms of mean absolute difference between achieved and planned HKA corrections, was 1.1° ± 1° (range, 0.0°-3.9°). The accuracy of femoral torsion correction, in terms of mean absolute difference between achieved femoral rotation and planned femoral rotation, was 2.4° ± 1.9° (range, 0.3°-7.5°).

## Discussion

The most important finding of this study was that a DFO utilizing PSIs can reliably be used to correct coronal and rotational deformities of the femur simultaneously. High accuracy was achieved for the correction of both coronal and rotational deformities, with absolute mean differences from planned to achieved correction of 1.1° and 2.4°, respectively. Accordingly, the hypothesis of this study could be confirmed.

DFO is an established surgical treatment option for correction of coronal deformities with concomitant unicompartmental degeneration or overload of the knee,^6,31^ and is also an effective surgical procedure for correcting femoral rotational deformities, which are frequently observed in patients with patellofemoral instability.^35^ However, some patients experience both coronal and rotational deformities of the femur.^7,12^ Nevertheless, performing 2 separate osteotomies for the correction of both deformities in such patients seems unreasonable. In spite of that, different studies showed that in femoral rotational osteotomy, differing the orientation of the osteotomy plane can affect not only the femoral rotation, but also the coronal alignment of the leg.^18,25^ Considering these findings, coronal and rotational deformities of the femur can be corrected by 1 osteotomy using an appropriate planning and surgical execution. The feasibility of such an osteotomy was already described in a cadaveric study.^13^ Using a conventional surgical technique, Deng et al^5^ showed in 13 patients favorable clinical and radiological outcomes after correction of coronal and rotational deformities using DFO combined with medial patellofemoral ligament reconstruction. Likewise, Hinz et al^11^ used a conventional surgical technique to correct coronal and rotational deformities using DFO in 14 patients and showed favorable clinical outcomes. However, in both studies, details on surgical accuracy are missing. Furthermore, a conventional surgical approach was used in both studies, but surgical navigation for such complex osteotomies may be favorable. Therefore, this study aimed to investigate the surgical accuracy of DFO for correcting coronal and rotational deformities of the femur using PSIs in a large cohort of patients with uniform pre- and postoperative radiological data.

The applicability and the beneficial use of PSIs in different osteotomies around the knee have already been proven.^8,20,22-24^ Furthermore, the use of PSIs has even been shown to result in more accurate correction compared with the conventional technique in isolated coronal realignment DFO,^16,32^ representing a less complex procedure compared with DFO for the simultaneous correction of coronal and rotational deformities. Considering the findings of this study, a high accuracy was found for coronal correction of the HKA, with a mean absolute difference between achieved and planned corrections of 1.1°. Even though a significant difference was found between planned and achieved coronal corrections (*P* = .018), 56% of cases showed a deviation from the achieved to the planned correction <1°, and the overall accuracy of 1.1° achieved in our study aligns with previously reported accuracies for PSIs in correcting isolated coronal malalignment and surpasses the surgical accuracy reported for the conventional technique.^16^ The accuracy for the rotational correction was also shown to be excellent, with a mean absolute difference between the planned and achieved rotational corrections of 2.4°, which also surpasses the accuracy demonstrated in the literature (4.8°).^23^ In addition, we found no significant difference between planned and achieved corrections (*P* = .771). However, in 17% of cases, the rotational correction deviated by ≥5° from the planned correction, and in 19% of cases, the coronal correction deviated by ≥2°. The main reason for the deviation in these cases was likely the soft tissue structures during reduction of the osteotomy. Nonetheless, for rotational correction, 83% demonstrated a deviation <5°, and for coronal correction 56% had a deviation <1° from the planned target, indicating high accuracy.

The present study should be interpreted in light of its potential limitations. The most obvious drawback is the lack of a control group, to compare the accuracy with that of a conventional technique group. However, superior accuracy compared with the conventional technique has already been demonstrated for less complex osteotomies around the knee,^16,28,32^ and therefore favorable results can be expected for the more complex DFO for coronal and rotational correction. Finally, another limitation is that the intraclass correlation coefficient (ICC) was not evaluated for the assessed radiological parameters in our study. The reason for this is that high ICCs for the parameters in question are already well documented in the literature, specifically the ICC for HKA, which has been reported as 0.996 (95% CI, 0.994-0.998) in 2D weightbearing LLRs, and that for femoral torsion, which has been reported as 0.957 (95% CI, 0.910-0.979) in CT measurements.^9,10,21^

## Conclusion

Coronal and rotational deformities of the femur can accurately be corrected simultaneously by a DFO utilizing PSIs. High accuracy was achieved for correction of both coronal and rotational deformities, with absolute mean differences from planned to achieved correction of 1.1° and 2.4°, respectively.
